# Bioinformatics Analysis of Transcriptomic Data Reveals Refined Functional Networks for the Self-Renewal of Mouse Spermatogonial Stem Cells

**DOI:** 10.1155/2018/5842714

**Published:** 2018-07-08

**Authors:** Min Wang, Wene Zhao, Fuqiang Wang, Xiufeng Ling, Daozhen Chen, Tao Zhou, Ying Wang

**Affiliations:** ^1^Centre for Reproductive Medicine, The Affiliated Wuxi Maternity and Child Health Care Hospital of Nanjing Medical University, Wuxi 214002, China; ^2^Analytical and Testing Center, Nanjing Medical University, Nanjing 210004, China; ^3^Department of Reproduction, The Affiliated Obstetrics and Gynaecology Hospital of Nanjing Medical University and Nanjing Maternity and Child Health Care Hospital, Nanjing 210004, China; ^4^Central Laboratory, The Affiliated Wuxi Maternity and Child Health Care Hospital of Nanjing Medical University, Wuxi 214002, China

## Abstract

Spermatogonial stem cells (SSCs) are exquisitely regulated to reach a balance between proliferation and differentiation in the niche of seminiferous epithelium. Several extrinsic factors such as GDNF are reported to switch the transition, activating various intrinsic signaling pathways. Transcriptomics analysis could provide a comprehensive landscape of gene expression and regulation. Here, we reanalyzed a previously published transcriptome of two cell types (standing for self-renewing and differentiating SSCs correspondingly). First, we proposed a new parameter, the expression index, to sort the genes considering both absolute and relative expression levels. Using a dynamic statistical model, we identified a list of 1119 candidate genes for SSC self-renewal with the best enrichment of canonical markers. Finally, based on interaction relations, we further optimized the list and constructed a refined network containing integrated information of interactions, expression alternations, biological functions, and disease associations. Further annotation of the 521 refined genes involved in the network revealed an enrichment of well-studied signaling pathways. We believe that the refined network could help us better understand the regulation of SSCs' fates, as well as find novel regulators or targets for SSC self-renewal or preservation of male fertility.

## 1. Introduction

Spermatogonial stem cells (SSCs) of the testis serve as a source pool for the continuous process of spermatogenesis and preserve fertility across nearly the whole lifetime of male mammals [1]. The small populations of SSCs are the ancestors of numerous differentiated and specialized cells including spermatogonia, spermatocytes, spermatids, and mature sperms [2]. Thus, SSCs are rarely found in the seminiferous epithelium of adult testis. However, to maintain their multipotency, SSCs are tightly regulated to reach a balance between self-renewal and differentiation [3]. Recent studies showed that SSCs could also be reprogrammed to become embryonic stem-like cells with pluripotency, which indicating this precious cell population may be applied in clinic for the treatment of male infertility and testicular cancers [4].

Previous studies have generally revealed the biological features for the self-renewal and development of mouse SSCs [3]. In summary, SSCs are located in the basal part of seminiferous tubules. The surrounding microenvironment (including basal membrane, sertoli cells, and peritubular myoid cells), termed as a niche, is of vital importance for the fate decision of SSCs. SSCs are attracted to the niche by *CXCL12* and mainly regulated by two growth factors for self-renewal: glial cell line-derived neurotrophic factor (*GDNF*) and fibroblast growth factor 2 (*FGF2*). Both *GDNF* and *FGF2* are successfully used to establish a system for long-term in vitro culture of self-renewing SSCs [5, 6]. However, the detailed molecular mechanisms for the regulation are not well elucidated.

Following extrinsic signal stimulations from the niche, it is believed that the intrinsic gene expression within the SSCs is consequently altered. Gene expression analysis based on high-thought technologies provides an efficient approach for initial screening of key regulators. Early in 2006, the Oatley et al. constructed the transcriptome of mouse SSCs under GDNF withdrawal using microarray [7]. This dataset provides a valuable resource for identifying important genes for the self-renewal and survival of SSCs. For example, several genes such as *Bcl6b*, *Etv5*, and *Lhx1* were further verified and studied using various functional experiments [8, 9]. Compared to microarrays, the recent emerging RNA-Seq technology has higher coverage and less noise, which enables the identification of more differentially expressed genes with high confidence [10, 11]. Recently, the gene expression profilings of SSCs, differentiating spermatogonia cells, meiotic cells, and haploid cells, were constructed using RNA-Seq technology [12, 13], providing abundant resources for studying the regulation of spermatogenesis at the gene level.

The main bottleneck of transcriptomic study is in the step of statistical and bioinformatics analyses. Usually, a list of candidate genes were generated using widely accepted statistical criteria (such as a combination of *P* value and fold change strategy). Then, automatical functional annotation based on knowledgebase, such as Gene Ontology (GO) and KEGG pathway, was performed to translate the gene list to biomedical significance [14]. We previously proposed a framework for reanalysis of published proteomics data to revise candidate protein list and dig novel findings [15]. And we believe that the reanalysis of transcriptomes using optimized bioinformatics methods could also help us to better interpret the data.

In the present study, we firstly extracted the expression data of two cell types (primitive type A spermatogonia versus type A spermatogonia, approximately standing for self-renewing and differentiating conditions in vivo) from a previously published dataset [12]. Then, we evaluated the expression features of eight canonical markers in RNA-Seq data. We also proposed a new parameter, the expression index, to integrate both absolute and relative expression abundances. Using this parameter, we developed a statistical model for dynamically screening the best cut-off considering the biological relevance. Finally, we constructed a refined network combining the information of physical interaction, expression change, biological function, and disease association, providing optimized and well-organized functional annotations for understanding and studying the maintenance of SSCs.

## 2. Materials and Methods

### 2.1. Data Collection and Processing

The quantification values of protein-coding genes were directly extracted from the calculated results based on fragments per kb of exon model per million mapped fragments (FPKM) in a previously published dataset [12]. The data of only two cell types were used: primitive SG-A (primitive type A spermatogonia) and SG-A (type A spermatogonia). We further filtered out low-quality data by requiring a minimal FPKM value of 0.1. The differentially expressed genes were identified using the Cuffdiff module embedded in the Cufflinks package (version: 2.2.1) [16]. For better representing the absolute abundance of gene expression, we calculated the percentile rank for each gene. In addition to fold change, we also used the average change percentage to represent the relative expression change. Then, the expression index was calculated by multiplying the average percentile rank by the average change percentage for each gene. The Pearson method was applied to analyze the correlation between the average abundances and fold changes of canonical markers.

### 2.2. Dynamical Prioritization of Candidate Genes

To optimize the selection of candidate genes highly associated with the corresponding research background, we developed a model which combines expert knowledge and statistical inference. First, we proposed to calculate a new parameter for each gene: the expression index (by multiplying the average change percentage by the average percentile rank). Thus, a higher expression index represents a more confident expression change. Then, the overall expression changes were ranked according to expression change. We further used two gene lists as expert knowledge (canonical genes for SSC self-renewal and genes annotated to be associated with cell proliferation or differentiation) to dynamically search for an optimized cut-off, which can generate a result with maximum positive genes. Fisher's exact test was used to compare the percentage of positive genes between dynamic selected genes and all identified genes. A *P* value less than 0.05 was considered for a statistically significant enrichment in the selected genes.

### 2.3. Functional Annotation and Network Analysis

Genes associated with cell proliferation or differentiation (in terms of biological process) were extracted from the GO database [17]. Phenotype information (including abnormal male infertility and abnormal spermatogenesis) based on mouse models were obtained from the Mouse Genome Informatics (MGI) database [18]. The protein-protein interaction relations of candidate genes were annotated using the STRING (version 10.5) database with a high confident cut-off score of 0.7 [19]. We further used the Cytoscape (version 3.2.0) software to reconstruct, analyze, and visualize the network. The interactions, biological functions, and phenotype associations were all integrated to generate a refined network. Pathways associated with signaling transduction were predefined by the Kyoto Encyclopedia of Genes and Genomes (KEGG) database [20]. Fisher's exact test was used to search for enriched pathways and biological processes in the refined network.

## 3. Results and Discussion

### 3.1. Expression Features of Canonical Markers for SSC Self-Renewal

**Figure 1:**
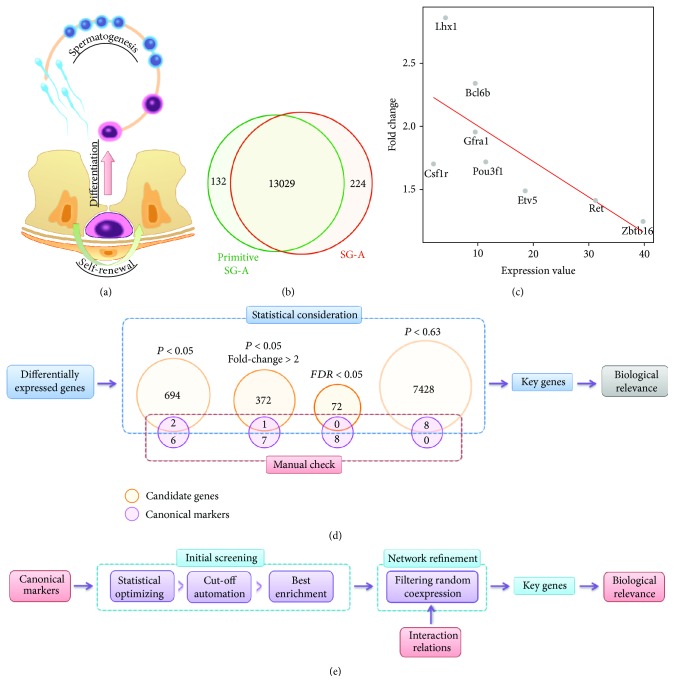
SSC fate decisions and expression features of transcriptomics data. (a) SSCs are well-regulated in the niche to maintain their multipotency as well as the capacity for continuous spermatogenesis. (b) Comparison of genes expressed in primitive SG-A and SG-A. (c) Correlation between fold change and expression level for canonical markers. (d) The traditional strategy for identifying candidate genes based on statistical consideration only. (e) The proposed optimized strategy for identifying key genes considering both biological relevance and statistical optimizing.

As described above, communicated to the niche factors, SSCs are strictly regulated to keep their multipotency and to continuously generate different stages of spermatogenic cells (Figure 1(a)). Till now, a few canonical markers, including *Bcl6b*, *Csf1r*, *Etv5*, *Gfra1*, *Lhx1*, *Pou3f1*, *Ret*, and *Zbtb16* (*Plzf*), are well-studied and known to play important roles in regulating SSC self-renewal [1, 21, 22]. The present study is aimed to screen a revised list of genes highly associated with the maintenance of the SSC pool. First, we chose a recently published gene expression dataset of multiple mouse spermatogenic cells quantified by RNA-Seq [12]. Since we focused on the self-renewal of SSCs, we only used the data from two cell types: primitive SG-A (are mostly SSCs) and SG-A (are mostly differentiating spermatogonial cells), which provides a paired model for analyzing the transition from self-renewal to differentiation in vivo. In summary, a total of 13,385 protein-coding genes were identified combining two cell types (Figure 1(b); Supplementary data 1). We then evaluated the absolute and relative expression features of the canonical markers. To better represent the absolute and relative abundance of gene expression, we calculated the average percentile rank and change percentage, respectively, for each gene. All eight canonical markers were upregulated in primitive SG-A, which is consistent with their theoretical change trend, indicating the high quality of RNA-Seq data.

The average percentiles range from 0.30 to 0.89, which indicates that most markers are highly expressed in SSCs. However, the values of fold change only range from 1.25 to 2.86. As shown in Figure 1(c), we further found that the average abundances of these genes were negatively correlated with fold change (*r* = −0.70 and *P* = 0.05). Thus, the higher the absolute expression, the lower the relative expression change, suggesting that only a certain amount of increase in gene expression is required for the maintenance of SSC self-renewal. However, it also should be noted that the isolated cells were a mixture of different cell types. The primitive SG-A cells contain both SSCs and gonocytes, while SG-A cells are mostly differentiating SG with a small proportion of stem cells. Thus, the detected fold change may be underestimated to some extent.

Traditional strategies for identification of differentially expressed (DE) genes were only based on statistical inference. Usually, a final combination of fold change and *P* value was used as a cut-off. However, it seems that the determination of such a cut-off was rather arbitrary [23]. And it is nearly impossible to generate reliable *P* values if there is only one replication in each group using the current statistical methods. As shown in Figure 1(d), using a loose statistical cut-off (*P* < 0.05), a total of 696 DE genes could be identified. However, only two canonical biomarkers were covered by the list. While the medium (*P* < 0.05 and fold change > 2.0) and strict (false discovery rate: FDR < 0.05) criterion were applied, the total number of DE genes and markers was further decreased dramatically. And to include all markers in the list, the cut-off of *P* value will be increased to 0.63, which identified a total of 7436 candidate genes. Thus, the candidate genes identified by statistical consideration only will lose the majority of canonical markers, resulting in a poor biological relevance. Here, we proposed a new bioinformatics approach, which combines expert knowledge and a dynamic statistical evaluation model, for screening of candidate genes with high biological relevance (Figure 1(e)). First, we will start from the canonical markers and develop a statistical model to enrich these markers. Then, we will also use interaction relations to further revise the list and to construct a refined regulation network. The traditional strategy was not sensitive to canonical biomarkers, possible due to technical and biological variation. We believe that the proposed new strategy will identify more markers and coexpressed genes, which will be more relevant to the research background.

### 3.2. Expert Knowledge-Guided and Dynamic Screening of Candidate Genes for Maintaining SSCs

**Figure 2:**
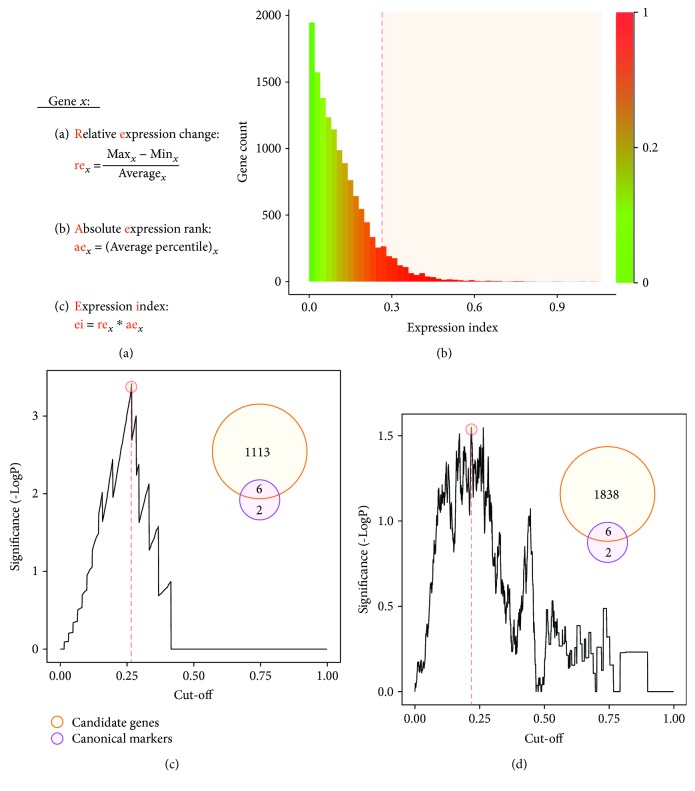
Dynamic screening of candidate genes. (a) The formula for calculating the expression index. (b) Distribution of gene count ranked by the expression index. (c) Dynamically optimizing the best cut-off using the canonical markers as positive reference (d). Optimizing the best cut-off using genes associated with cell proliferation or differentiation as positive reference.

First, we created a new parameter for comprehensively evaluating the overall confidence of expression change: the expression index, which is equal to the average change percentage multiplied by the average percentile rank for each gene (Figure 2(a)). The average change percentages mapped all fold changes to the range of 0 to 2, which solves infinite values of expression change (absent change will convert to a value of 2). However, some values of large fold changes were unreliable due to low abundance issue. For example, a gene increase from 0.1 to 0.2 (2 folds) is apparently less confident than an increase from 100 to 200. Thus, we used the value of the average percentile rank to normalize the change trend, which could be used to filter out those low values as well as to bubble up confident changes (Figure 2(b)). After applying this algorithm, the canonical markers ranged from 0.16 to 0.41.

We chose the canonical markers as expert knowledge to optimize the screening of candidate genes. We created a statistical model which dynamically evaluates the enrichment of positive reference genes (canonical markers). By an increment value of 0.001, we tested the results of identifying positive genes using all possible cut-off values of the expression index. As shown in Figure 2(c), as the expression index increases, the significance of enrichment rises and then falls as expected. Contrary to the traditional statistical inference using an empirical cut-off, our mission is to dynamically find an optimized list of coexpressed genes with maximum positive results as well as minimum negative results based on expert knowledge. According to the results, the best cut-off was an index value of 0.267 (*p* < 0.001), which identified six markers in 1119 candidate genes. And the best cut-off to include all markers was 0.156 (*p* = 0.010), identifying a total of 3585 genes.

The selected pair of cell types stands for a transient status from self-renewing to differentiating. Thus, differentially expressed genes between these two groups should be highly associated with cell proliferation and differentiation theoretically. Then, we also tested another model to use all genes associated with cell proliferation or differentiation as positive genes (Figure 2(d)). A total of 3517 genes were annotated to be associated with cell proliferation or differentiation based on GO annotations (including all of the eight canonical markers). Similarly, the best cut-off was 0.218 (*P* = 0.028), which finds a total of 1844 differentially expressed genes with 547 genes associated with cell proliferation or differentiation (Supplementary data 2). Among these genes, six canonical markers were also included. And the best cut-off to include all markers was 0.121 (*P* = 0.045) with a cost of incorporating 5037 genes. Overall, the presented dynamic model using the expression index provides a simple and robust strategy for the initial screening of candidate genes directly associated with the research background. This strategy could also be applied in other omics data (for both transcriptomics and proteomics analyses) to prioritize the gene list and find an optimized cut-off in experiments with or without biological replications.

### 3.3. Construction of a Refined Expression-Function Relation Network

**Figure 3:**
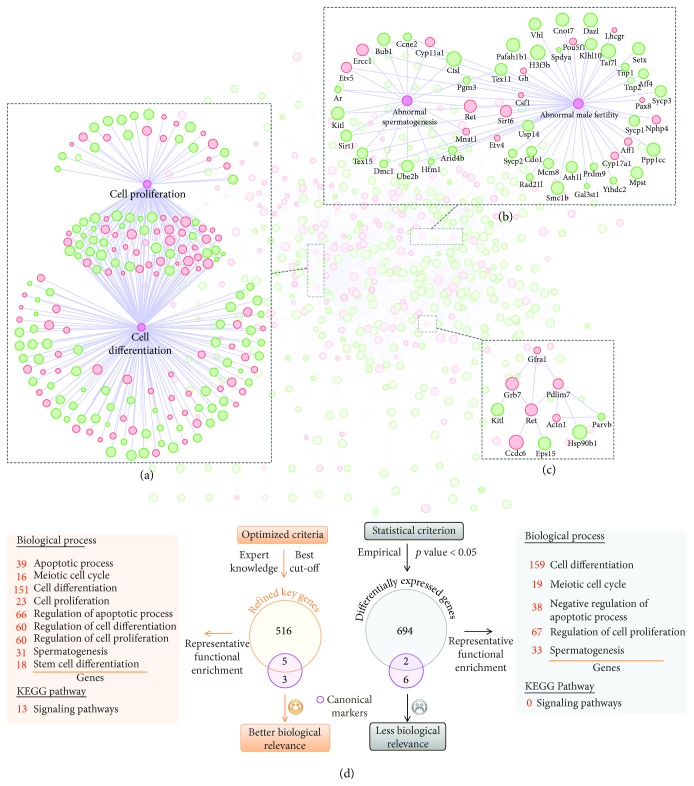
The refined expression-function network. (a) Subnetwork for genes involved in cell proliferation or differentiation. (b) Subnetwork for genes associated with abnormal male infertility or spermatogenesis. (c) Subnetwork for Gfra1-centric relations (extended to two neighboring levels). Red or green color represents up- or downregulation in primitive SG-A. The inner circle size and the border width indicate the absolute and relative expression levels, respectively. (d) The detailed comparison of the biological relevance between the refined and traditional DE gene lists.

Balanced between self-renewal and differentiation, SSCs were tightly regulated by various extrinsic signal factors in the niche. Starting from the membrane receptors, a series of intrinsic genes were thought to be activated or silenced. Thus, it is important to organize these genes in a network for better understanding their regulation relationships and cascade signaling transductions. In addition, the initial list generated above may contain irrelevant coexpressed genes, which share similar expression patterns with canonical markers but not functionally associated. Using the 1119 candidate genes identified above, we first searched for potential interactions among these genes using the STRING database [19]. In total, we generated a complex relation network containing 521 genes with 1149 pairs of relations based on known and predicted protein-protein interactions with high confidence. To better analyze and visualize this network, we used the Cytoscape software to reconstruct the network. We also anticipated that a well-organized network could help us understand the dynamic landscape of gene regulation as well as find novel regulators for the self-renewal of SSCs. Thus, we mapped the absolute and relative expression information to the network, indicating the change trend and confidence of expression alternation. We also searched for all known genes associated with male fertility (based on the phenotype data of MGI) in addition to the functional terms of cell proliferation and differentiation. Combining the information of interaction, expression, biological function, and disease association (mouse phenotype), we finally generated a refined network for interpreting the self-renewal of SSCs at the gene level (Supplementary data 3). In summary, a total of 192 genes were upregulated in self-renewing SSCs, while 329 genes were downregulated. For functional annotation, 196 (37.6%) genes were found to be involved in cell proliferation or differentiation (Figure 3(a)), and 54 (10.4%) genes were proven to cause phenotypes of male infertility or abnormal spermatogenesis (Figure 3(b)). The percentages of the two classes of genes in the network are significantly greater than those in all identified genes (26.3% and 4.6%, resp.), further indicating that the refined network is highly related to the corresponding research background.

GDNF-dependent signaling transduction is a classical pathway responsible for the maintenance and self-renewal of SSCs both in vivo and in vitro [24]. Among the refined network, five of the eight canonical markers (*Etv5*, *Gfra1*, *Lhx1*, *Pou3f1*, and *Ret*) were involved, indicating that the predicted network may cover a wide range of bona fide regulatory interactions associated with SSC self-renewal. For example, *Gfra1* is the direct receptor of GDNF, which is located on the cell surface, and *Ret* (a tyrosine kinase transmembrane) binds GDNF and triggers the activation of multiple intrinsic signaling pathways [25]. One of the most studied signal pathways involved in SSC self-renewal was the PI3K-Akt pathway [26]. As indicated in Figure 3(c), the direct or cascaded interactions of *Gfra1*-*Ret*-*Kitl*/*Hsp90b1* (Kitl and *Hsp90b1* are key genes in the PI3K-Akt pathway) were prioritized in the Gfra1-centric subnetwork. Based on KEGG database, we further searched for potential regulating signaling pathways in the refined network. As listed in Table 1 (Supplementary data 4), a total of 13 pathways were enriched, containing the most studied pathways for the maintenance of SSCs including MAPK, PI3K-Akt, Ras, and Wnt signaling pathways [21, 27].

In addition to prioritization of well-known genes, we suggested that the refined network could also be used to find novel regulators for SSC self-renewal. For example, in a previous proteomic study, with the help of biological annotation and network construction, we successfully identified *Raptor* as a downstream regulator for GDNF-dependent cell proliferation [28]. However, it should also be noted that the present network was derived from RNA-Seq data. Many signaling pathways are highly regulated at protein level with the alternation of post-translational modification such as phosphorylation. Thus, the present network may be limited for identifying pivotal genes associated with phosphorylation. However, we believe that this could be improved by integration of transcriptomics and proteomics data under similar conditions. Besides, patients with testicular cancer may lose the ability to generate germ cells following anticancer treatments. More and more studies started to establish models of SSC transplantation for restoring male fertility [29, 30]. Since the self-renewal of SSCs is the foundation of continuous spermatogenesis, the network may also help us in identifying target genes for the preservation of male fertility.

Finally, we comprehensively compared the technical features and biological relevance between the refined and traditional DE gene lists (Figure 3(d)). The present optimized strategy used canonical markers as true positive genes to automatically find the best cut-off of the expression index and generate a list of candidate genes (including 5 of the 8 markers). However, the traditional approach only considered statistical issue. The cut-off was based on the empirical *P* value of 0.05, and only two canonical markers were identified. We also performed functional enrichment (in terms of biological process and KEGG pathway) analyses using these two gene lists. The refined gene list enriched many representative functional terms including apoptosis, cell cycle, cell differentiation, cell proliferation, spermatogenesis, and various signaling pathways. Although the *P* value-derived list can also identify a few terms about cell cycle, apoptosis, cell differentiation, cell proliferation, and spermatogenesis, no signaling pathways were enriched. There are two main reasons that the refined gene list obtains better biological relevance. First, the refined list enriched more coexpressed genes using canonical biomarkers as a positive reference. Second, some coexpressed genes without functional associations were further removed based on the interaction network.

## 4. Conclusions

Although transcriptomics technology can provide a profiling of the entire gene expression and regulation, bioinformatics analysis is a critical step for translating the gene list to biomedical significance. Traditional screening of candidate genes among two groups is usually based on statistical inference using a one-size-fits-all cut-off. In the present study, we first ranked the genes considering both absolute abundance and relative change, by the proposed expression index. Then, taking well-studied genes (known to be associated with SSC self-renewal) as the positive reference, we constructed a statistical model which dynamically screens for the best cut-off to prioritize candidate genes. This model was further verified using predicted genes involved in cell proliferation or differentiation as positive genes, providing a simple and robust approach to find an optimized cut-off for identification of functional important genes with minimal false discovery rate.

Triggered by exogenous factors secreted by the surrounding cells, various endogenous genes are thought to be activated or silenced for maintaining the proliferation and survival of SSCs. Although a few key regulators and signaling pathways are reported, a well-organized level of annotation is required to provide a comprehensive understanding of the mechanism of SSC self-renewal in vivo. Here, we chose two cell types (primitive SG-A versus SG-A) as a transient model of self-renewing versus differentiating. By reanalyzing the comparative transcriptome of these cells, we identified a list of 1119 candidate genes with best enrichment of canonical markers using the expert knowledge-guided and dynamic statistical model as mentioned above. Using these genes, we finally constructed a refined network combining information of physical interaction, expression change, cellular function, and disease association. This network contains five of the eight canonical markers and also enriches the most important signaling pathways, indicating a high quality and relevance of gene prioritization. And we suggested that the refined network could also be used to find novel regulators for SSC self-renewal, as well as target genes for treatment of male infertility or testicular cancers.

## Figures and Tables

**Table 1:** Enriched signaling pathways in the network.

Pathway ID	Pathway name	Gene count	*P* value
4010	MAPK signaling pathway	21	2.7*E* − 05
4151	PI3K-Akt signaling pathway	21	8.5*E* − 04
4014	Ras signaling pathway	16	8.9*E* − 04
4310	Wnt signaling pathway	11	4.8*E* − 03
4022	cGMP-PKG signaling pathway	12	5.2*E* − 03
4668	TNF signaling pathway	9	7.7*E* − 03
4621	NOD-like receptor signaling pathway	6	1.2*E* − 02
4012	ErbB signaling pathway	7	1.8*E* − 02
4015	Rap1 signaling pathway	11	4.1*E* − 02
4068	FoxO signaling pathway	8	5.0*E* − 02
4921	Oxytocin signaling pathway	12	3.0*E* − 03
4261	Adrenergic signaling in cardiomyocytes	10	1.2*E* − 02
4915	Estrogen signaling pathway	7	2.8*E* − 02

## Data Availability

The source expression data was extracted from a previously published dataset, which can be obtained from the Gene Expression Omnibus under accession GSE35005.

## Supplementary Materials

Supplementary Materials Supplementary data 1: detailed list for gene expression and annotation. Supplementary data 2: detailed results of dynamic screening of candidate genes. Supplementary data 3: detailed information for the refined network. Supplementary data 4: detailed results of functional enrichment analyses.
